# Development of a valid Chinese version of the Cumberland Ankle Instability Tool in Chinese-speaking patients with chronic ankle instability disorders

**DOI:** 10.1038/s41598-021-87848-x

**Published:** 2021-05-07

**Authors:** Wei Wang, Dongfa Liao, Xia Kang, Wei Zheng, Wei Xu, Song Chen, Qingyun Xie

**Affiliations:** Department of Orthopedics, The General Hospital of Western Theater Command, Tianhui Road 270, Chengdu City, 610000 People’s Republic of China

**Keywords:** Psychology, Quality of life, Outcomes research

## Abstract

As an effective scale for the condition assessment of patients with chronic ankle instability (CAI), the Cumberland Ankle Instability Tool (CAIT) is the most widely used scale, and its original version is written in English. Therefore, the purpose of our study is to apply the CAIT to Chinese patients and evaluate its responsiveness, reliability, and validity in terms of Chinese patients with CAI. First, we adapted the CAIT into the Chinese edition (CAIT-C), through which cross-cultural adaptation and translation can be carried out in a five-step procedure. Next, recruited patients completed the three periods of the Foot and Ankle Ability Measure (FAAM), CAIT-C, and the Medical Outcomes Study Short-Form 36 (SF-36) scales. Afterward, to assess the responsiveness, reliability, and validity, we calculated the standardized response mean (SRM), effect size (ES), Spearman's correlation coefficient (*r*_s_), minimal detectable change (MDC), standard error of measurement (SEM), intraclass correlation coefficient (ICC), and Cronbach’s alpha. Generally, in the use of CAI, 131, 119, and 86 patients favorably completed the three periods of the scales. The CAIT-C was proven to have good test–retest reliability (ICC = 0.930) and fine internal consistency (Cronbach’s alpha = 0.845–0.878). The low-value of MDC (0.04–2.28) and SEM (1.73) show it is possible to detect clinical changes when we take advantage of CAIT-C. Good or moderate correlations (*r*_s_ = 0.422–0.738) were gained from the physical subscales of the SF-36 and the subscales of the FAAM and the CAIT-C. Fair or poor correlations (*r*_s_ = 0.003–0.360) were gained between the mental subscales of the SF-36 and the CAIT-C, which sufficiently indicated that the CAIT-C had good validity. Moreover, good responsiveness was observed in the CAIT-C (ES = 1.316, SRM = 1.418). The CAIT-C scale is an effective, valid, and reliable tool to evaluate Chinese CAI patients.

## Introduction

The ankle joint is most vulnerable in daily activities as an important weight-bearing joint in the human body. The risk of repeated injury often increases greatly after the first ankle joint sprains^1^. Repeated sprains and instability of the ankle joint are in reciprocal causation, and at least 30% of ankle sprains will develop chronic ankle instability (CAI)^2,3^. The common symptoms of CAI are persistent pain in the ankle joint, repeated sprains, and recurrent “muscle weakness”^4^. Young athletes are more at risk of getting the initial injury^5^. However, chronic CAI impacts a wide age group of people, including those who have quit sports activities entirely^6,7^. According to relevant reports, the number of ankle sprains in the United States is as high as 23,000–27,000 per day^8^. Currently, the prevalence of CAI in the Chinese population has not been reported with high reliability. However, due to the large population of Chinese people, China may have a large number of CAI patients suffering from ankle sprains.

The greater prevalence of CAI and its impact on patients’ quality of life has led medical researchers and workers to focus more on diagnosing and treating this disease. Patient-reported outcome measures (PROMs) are among the most important tools for researchers to conduct relevant studies^9^. Since the 1980s, numerous studies have been carried out to develop patient-reported outcome measures (PROMs)^10^. The PROMS customarily collects relevant data in the form of an independent questionnaire. Through these questionnaires, doctors can have more acquaintance with the severity of the patient's state and provide more advisable treatment for the patient^11^. PROMs, which feature high efficiency, low costs, and good reliability, have been a subject of interest in research and clinical practice^12^.

The advantages of PROMs mentioned above make them widely applied in various groups of patients. In accordance with the goal, we can divide PROM into specific scales and generic scales. Specific scales can be applied to specific patients. For example, the Western Ontario Shoulder Instability Index (WOSI) is suitable for patients with unstable shoulder joints^13^, the Foot and Ankle Ability Measure (FAAM) is appropriate for diverse neuromuscular skeletal changes in the ankle/foot^14^, and the Cumberland Ankle Instability Tool (CAIT) is fitting for CAI^15^. The latter is used to assess the sufferers' general state, for instance, the most ordinary Medical Outcomes Study Short-Form 36 (SF-36).

CAIT is one of the most widely used and reliable PROMSs for CAI patients. As recommended by the National Athletic Trainers’ Association (NATA) and other professional organizations, PROMs can be used to identify how patients perceive ankle instability, thus helping to make treatment decisions during the management of CAI^16^. Hiller et al. developed the CAIT^15^, a discriminative scale used to identify CAI patients and evaluate the severity of functional ankle instability. CAIT is used in various countries worldwide for its ease of use, proven validity, and reliability. The International Ankle Consortium suggests adopting CAIT and other reliable and valid questionnaires to examine ankle instability self-reported by patients^17^.

Like most other typical PROMs, CAIT was originally written in English. If there were no language or cultural differences, it could have been used worldwide. When patients from different cultural backgrounds are treated with a reliable and effective scale, it is vital to test the psychometric properties of the scale instead of simply translating content to avoid assessment deviation secondary to cultural differences^18,19^. To apply CAIT to more people with CAI who speak different languages and have different cultural backgrounds, it has been compiled in six different languages (Japanese, Persian, Dutch, Spanish, etc.) by many studies^11,20–25^. Although a previous study complied and translated it into Chinese^26^, it lacks an analysis of the validity of the scale, which is the most important and necessary psychometric assessment. In addition, whether the subjects were right for the study has yet to be identified (ordinary people rather than patients with CAI were selected). As a result, we think it is necessary to compile CAIT more accurately and systematically across cultures, translate it into Chinese, and apply it to the largest number of CAI patients^27,28^.

Thus, we aimed to translate CAIT into the Chinese Version (CAIT-C) and assess the responsiveness, reliability, and validity of the CAIT-C in CAI patients.

## Methods

### Translation and cross-cultural adaptation

The principles of previously published guidelines were followed to translate the CAIT from the original version^10,29^. The whole process was composed of five steps. The specific contents have been detailed in a similar article published in our previous publication^30^.

### Patients and data acquisition

Consecutive native patients who had CAI, spoke Chinese, and visited Chengdu Military General Hospital from February 2016 to March 2018 were enrolled in this study. The inclusion criteria were as follows: (1) age > 18 years with independent signing authority and (2) they reported no less than two cases of severe ankle sprains and a series of feelings including chronic pain, ankle instability, and/or “giving way” in daily life or sports activities. The exclusion criteria were as follows: (1) previous surgical musculoskeletal structures and fractures requiring readjustment in the history of the lower extremity limbs; (2) severe injury to the musculoskeletal structures of the lower limb joints over the past three months; and (3) other chronic inflammatory diseases in the lower limbs that might impact ankle function. Patients who satisfied these criteria and were willing to participate in this study remained under the premise that the sample capacity standard for PROM research was put forward by Terwee et al.^14^. More than one hundred patients’ questionnaires were used for internal consistency analysis, and more than fifty patients’ questionnaires for ceiling or floor validity, effects, and reliability analysis. All participants read and signed the informed consent form approved by our ethics committee (Chengdu Military General Hospital).

On the first day of admission to the hospital, the patients were required to offer demographic information and, in a quiet meeting room, complete four scales independently. The SF-36, CAIT-C, FAAM, and SC-IdFAI (for another study) were included. One day before the beginning of physiotherapy, which was 1 week after the first set of scales, they completed the CAIT-C for the second time to assess the scale of test–retest reliability. Patients were excluded if they had related treatment in the previous week. Finally, patients who voluntarily received 8 weeks of physiotherapy at our hospital completed the CAIT-C for the third time following therapy to assess responsiveness.

### Scales

The CAIT comprises nine items with multiple options related to different aspects of CAI, such as ankle pain, subjective instability during daily and physical activities, and the ankle’s response to episodes of giving way^24^. The nine items generate a total score ranging from 0 to 30, with lower scores indicating more severe instability and 30 as the best possible score. The original study established a cutoff score of ≤ 27 to identify those with CAI^15^.

The FAAM is a region-specific scale designed to assess the function of the foot and ankle^31^. It consists of two subscales: activities of daily living (ADL) and sports. The ADL subscale and sport subscale score ranges are 0–84 and 0–32, respectively. The higher the score, the better the functional status. The FAAM is a region-specific scale rather than a disease-specific scale; however, it has been proven to have good validity in patients with CAI^32^. The SF-36 is a common quality of life evaluation scale, and 8 subscales of 35 items were included. It can assess a patient’s state, including social function, mental health, and physiological function. Each subscale of the SF-36 has its particular marking method, and the ultimate score is changed to a 100-point system. Similarly, the patient's quality of life, or functional status, is increased with increasing scores^33^. The abovementioned scales have been translated into Chinese versions, and it has been proven that these editions are of great responsiveness, reliability, and validity^34,35^.

### Psychometric assessments and statistical analysis

Reliability is the degree to which a measurement is free from error^36^. The reliability tests of CAIT-C chiefly contain measurement error, internal consistency, and test–retest reliability. The degree of internal consistency is described as the degree of interaction among projects^35^, which is chiefly assessed by the scale of Cronbach's α value of the scale. When α > 0.7, 0.8, and 0.9, the scale has acceptable, good, and excellent internal consistency, respectively^14^. However, extremely high values of Cronbach’s α (> 0.95) also show item redundancy^37^. Additionally, Cronbach's α was calculated for the CAIT-C, so if an item was removed, one could see if the item negatively influenced Cronbach's α^14,38^. The test–retest reliability of the scale is assessed in comparison with the previous two responses of patients to CAIT-C. The intraclass correlation coefficient (ICC), rooted in the two-way analysis of the variance in a random effect model, is its assessment indicator. Once ICC > 0.9 and 0.8, the scale has excellent and good reliability, respectively^39^. To observe the systematic errors between surveys, we have further depicted the Bland–Altman diagram^40^. The measurement error includes the randomness and systematic error, which the patient scores, and has nothing to do with the real change of the structure tested^41^. It was calculated in accordance with the formula and analyzed using the standard error of measurement (SEM): SD × √ (1 − ICC). In the first evaluation, the standard deviation of all patients was expressed in SD^41^. The minimal detectable change (MDC) reflects the minimum individual change of fraction, which can be understood as a real change. It was calculated as SEM × 1.96 × √2/√n at the group level and SEM × 1.96 × √2 at an individual level^42^.

We can evaluate the validity of the CAIT-C through its construct validity and content validity. The evaluation of the relevance of the items and comprehensiveness is contained in content validity^43^. The three project comprehensive evaluation indexes are patients’ feedback, the response rate, and ceiling/floor effects. Assuming that the ceiling/floor effect is lower than 15%, the feedback of the patients is more than 95%, and the patients in the filling scale have no feedback on the difficulty of understanding, then the judgment scale has great comprehensiveness^14,44^. In addition, we invited one rehabilitation specialist and two orthopedic specialists to help judge whether the items were relevant for the construct to be measured and for the patients with CAI^43^. Since the gold standard for assessing CAIT-C standard validity does not exist, the hypothesis test is used to assess the construct validity of CAIT-C. Construct validity is the extent to which the scores on a scale are consistent with hypotheses based on the assumption that the scale validly measures a specific construct^43^. In this study, we selected the FAAM and SF-36 as the control scales for the CAIT-C. Based on the contents of each scale, we assumed that the CAIT-C might have good correlations with the physical subscales of the SF-36 (physical functioning, role physical, bodily pain, general health) and FAAM, and poor correlations with the mental subscales of the SF-36 (vitality, social functioning, role emotional, mental health). In addition, we assumed that the correlations between CAIT-C and FAAM might be stronger than those between CAIT-C and SF-36. Details of the relevant hypotheses among the abovementioned scales are shown in Table 4. Based on the above hypotheses, we calculated Spearman’s correlation coefficient (*r*_s_) between the CAIT-C, SF-36, and FAAM by using the results of the patients’ first fill-out scale and evaluated the construct validity of the CAIT-C by comparing the consistency between these data and the hypotheses detail. Good construct validity was based on meeting the criterion for at least 75% (8/10 or more) of stipulated a priori hypotheses^41^. The correlations were judged as excellent (*r*_s_ = 0.8–1.0), good (*r*_s_ = 0.6–0.8), moderate (*r*_s_ = 0.4–0.6), fair (*r*_s_ = 0.2–0.4), or poor (*r*_s_ = 0–0.2)^30^.

Responsiveness is a scale to detect the capability of the structure to be measured over time^43^. We compared the results of the 8-week scale before and after physiotherapy to assess the responsiveness of CAIT-C. The two indicators of reactivity evaluation are standardized response mean (SRM) and effect size (ES). We define the SRM by dividing the average change between each time point by the SD of this change. ES indicated the average change in treatment outcome within 8 weeks before and after the operation, divided by the SD of CAT-C before treatment^45^. When the SRM and ES values exceed 0.80, they are large; when the values are between 0.51 and 0.80, they are intermediate; and when they are less than 0.50, they are small^46^.

Statistical Package for the Social Sciences, version 20.0 (SPSS, Chicago, IL, USA), was utilized for statistical analysis. The data are expressed as the mean and standard deviation (SD). The 95% confidence intervals (CIs) can report the ICC value. A P value less than or equal to 0.05 was considered statistically significant.

### Ethical statement

All procedures performed in this study involving human participants were carried out in accordance with the 1964 Helsinki declaration and its later amendments or comparable ethical standards. All participants read and signed informed consent, and this clinical study obtained the approval of the ethics committee of our hospital (The General Hospital of Western Theater Command).

## Results

### Patients

A total of 161 patients with CAI (104 males and 57 females) who came to our hospital from February 2016 to March 2018 met the screening criteria. In the end, 132 patients (82% of the invited, 46 women, and 86 men) were invited to participate, and all patients completed the scale. One week later, 119 patients (81 males and 38 females) completed CAIT-C for the second time through reexamination in our hospital, an inquiry by telephone, or emails. Of the 13 patients who did not complete the second questionnaire, nine patients were excluded because they had received the relevant treatment (physical therapy or analgesic drugs) in the previous week, and four patients were out of contact. In addition, 86 of all patients (104 males and 57 females) received regular physicotherapy in our hospital, and they completed CAIT-C for the third time after all treatments were completed (8 weeks later). Thus, to evaluate the validity of the CAIT-C, measurement error, retest reliability, and internal consistency, we selected 132 samples, of which 119 samples were used to evaluate the retest reliability of the CAIT-C, and 86 cases were used to evaluate CAIT-C reactivity. Table 1 shows the detailed demographic data of the primary participants.

**Table 1:** Demographic and clinical characteristics of participants.

Characteristics	Number (%) or Mean ± SD
**Age (years)**	26.5 ± 5.7
Range	18–47
**Age groups**	
≦ 20	23 (17.4%)
21–30	81 (61.4%)
31–40	25 (18.9%)
≧ 41	3 (2.3%)
**Gender**	
Female	46 (34.8%)
Male	86 (65.2%)
**Affected side**	
Right	98 (74.2%)
Left	34 (25.8%)
Bilateral	
BMI (kg/m^2^)	23.4 ± 4.9

This study and the manuscript with DOI number “10.1186/s12891-020-03314-1” published by the same research team used the same cohort of Chinese CAI patients.

*BMI* body mass index.

### Translation and cross-culture adaptation process

The translation of CAIT, forward and backward, was very smooth. Since it was easy to comprehend the items of CAIT, we had not improved them. Twenty patients (10 women and 10 men) completed the final version of the CAIT-C in CAI patients. No patient indicated that the project was difficult to understand or that it lacked standardization.

### Reliability

The Cronbach’s α for the CAIT-C was 0.873, providing good internal consistency. Moreover, suppose that the Cronbach’s α coefficient of each item was deleted, as shown in Table 2, the correlation coefficient between each item's score and the remaining total score. In the analysis of the project, no improvement was found every time the items were deleted from the scale, except for items 8 and 9. When omission was omitted, the project increased slightly.

**Table 2:** The internal consistency of CAIT-C.

Items	Corrected item: total correlation^a^	Cronbach’s α if item was deleted
Overall scale	1.000	0.873
Item 1	0.762	0.858
Item 2	0.809	0.847
Item 3	0.756	0.854
Item 4	0.758	0.853
Item 5	0.710	0.861
Item 6	0.727	0.857
Item 7	0.826	0.845
Item 8	0.503	0.877
Item 9	0.537	0.878

*CAIT-C* Chinese version of Cumberland Ankle Instability Tool.

^a^Calculated by the Spearman’s correlation coefficient of the items with total score.

**Figure 1:**
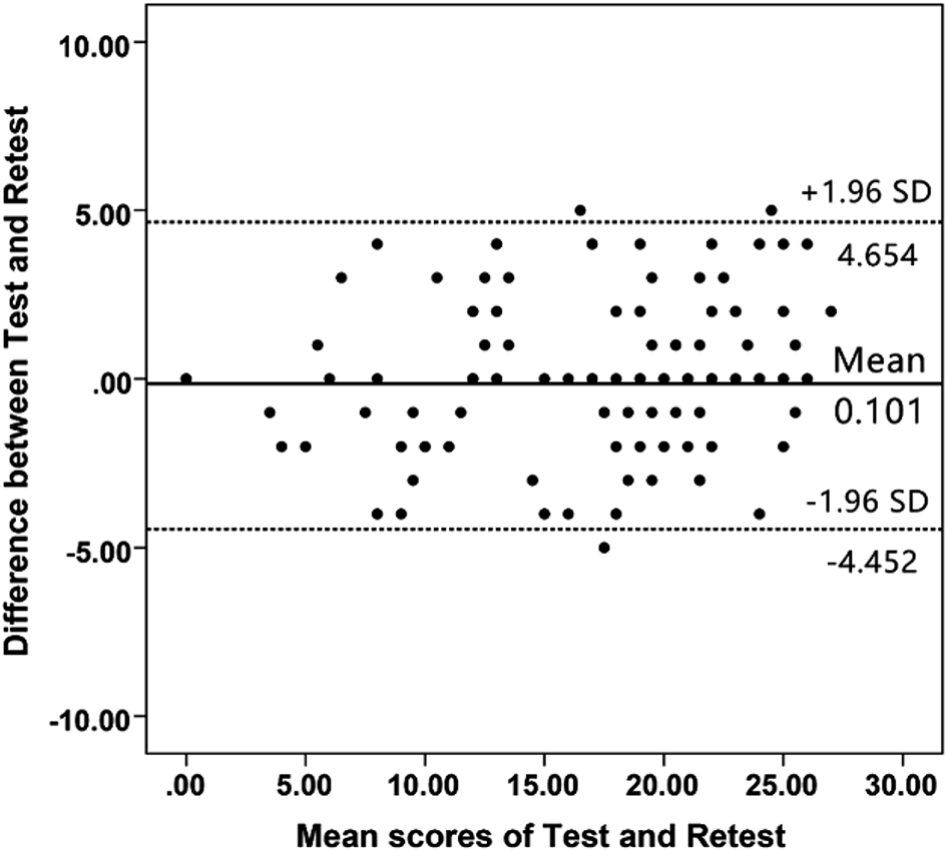
Bland–Altman plots of the test–retest reliability of the CAIT-C. Each data point indicates how the difference between the two test sessions for an individual patient compares to the mean of the two sessions for scores of each CAIT-C. The interval of two sessions was 1 week. The dashed line shows the 95% (± 1.96 SD) limits of agreement.

The ICC value of CAIT-C was 0.930, indicating that CAIT-C had excellent test–retest reliability (Table 3). In addition, Bland–Altman plots showed no systemic error in the first two rounds (Fig. 1), which confirmed that CAIT-C had a good test–retest agreement.

**Table 3:** The floor/ceiling effects, test–retest reliability, measurement error and responsiveness of CAIT-C.

	Current study	Dutch version	Persian version	Korean version	Portuguese version	Spanish version	Original version
Floor effect^a^	1.5%	1%	2.6%	–	0%	0%	–
Ceiling effect^a^	3.8%	2%	5.1%	–	7.9%	9%	–
ICC (CI range)	0.930 (0.901–0.951)	0.943 (–)	0.91–0.95 (0.80–0.97)	0.94 (–)	0.95 (0.93–0.97)	0.95–0.98 (0.93–0.99)	0.96 (–)
SEM	1.73	0.82	2.03–2.40	1.72	–	–	–
MDC (I)^b^	4.80	2.28	5.6–6.5	–	–	–	–
MDC (G)^c^	0.44	0.04	–	–	–	–	–
ES	1.316	–	–		0.75	0.69–1.07	–
SRM	1.418	–	–		–	–	–

*CAIT-C* Chinese version of Cumberland Ankle Instability Tool, *ICC* intraclass correlation coefficient, *CI* confidence interval, *SEM* standard error of measurement, *MDC* minimal detectable change, *ES* effect size, *SRM* standardized response mean.

^a^Percentage of patients with the worst (floor effect) and the best (ceiling effect) score.

^b^The MDC value at an individual level.

^c^The MDC value at the group level.

The SEM value of CAIT-C was 1.73. Therefore, the MDC reflecting the minimal individual and group (this study) change in score that can be interpreted as a real change was 4.80 and 0.44.

### Validity

In this research, there was no error in response to the CAIT-C questionnaire. The distribution of scores indicated there was no floor effect (1.5%) or ceiling effect (3.8%) in the CAIT-C (Table 3). In addition, no patient indicated that the contents of CAIT-C were difficult to understand. According to the assessment and analysis of two departments of orthopedics experts and rehabilitation experts, the amount of information obtained from each CAIT-C project is sufficient to assess the health-related quality of life of CAI patients. Hence, it is not recommended to remove or add any items. According to the above results, the CAIT-C has good content validity.

Table 4 shows the relevant data of the CAIT-C construct validity evaluation. It showed good (*r*_s_ = 0.624 to 0.738) correlations between the two subscales of the FAAM and CAIT-C, moderate (*r*_s_ = 0.422–0.560) correlations between the physical subscales of the SF-36 and CAIT-C, and fair or poor (*r*_s_ = 0.080–0.260) correlations between the mental subscales of the SF-36 and CAIT-C. The above results were completely consistent with our a priori hypotheses (10/10).

**Table 4:** Construct validity of the CAIT-C.

Scales	Spearman’s correlation coefficient (*r*_*s*_) ^a^	*P* value	Hypotheses
**FAAM**			
ADL	0.738	< 0.0001	≥ Moderate, and better than SF-36 wih CAIT-C
Sport	0.642	< 0.0001	
**SF-36**			
Physical function	0.443	< 0.0001	≥ Moderate, and worse than FAAM wih CAIT-C
Role-physical	0.560	< 0.0001	
Bodily pain	0.522	< 0.0001	
General health	0.422	< 0.0001	
Vitality	0.260	0.003	≤ Poor, and worse than physical subscales of SF-36 and CAIT-C
Social function	0.140	0.109	
Role-emotional	0.080	0.360	
Mental health	0.183	0.036	

*CAIT-C* Chinese version of Cumberland Ankle Instability Tool, *FAAM* foot and ankle ability measure, *ADL* activity of daily living, *SF-36* Short-Form 36.

^a^Calculated by the Spearman's correlation coefficient (*r*_*s*_) of the CAIT-C with FAAM and SF-36.

### Responsiveness

The questionnaires were completed before and after physicotherapy to assess the responsiveness of CAIT-C, and the relevant data is listed in Table 3. Overall, the average CAIT-C score increased after treatment. The values of SRM (1.418) and ES (1.316) are both greater than 1, which suggests that CAIT-C has good responsiveness.

## Discussion

In clinical surveys, PROMs are tools of great importance. Researchers can compare the questionnaire reports from similar studies and quantify the functional condition of patients. This is very helpful for an increasing number of multicenter clinical studies^10^. Today, in China, clinical research is developing rapidly, and many relevant papers are published every year. This is because there is a large number of patient groups in China and also because of the government’s emphasis on scientific research^47^. Therefore, China is now in great need of effective PROMs. These scales can help many patients in China receive a more accurate diagnosis and treatment and provide support for many clinical studies in China.

CAIT is one of the most widely used PROMs for CAI patients. Only one study has reportedly performed the cross-cultural translation of CAIT in Chinese^26^, but the validity indexes of CAIT-C, the most important part, were not evaluated in that study. There was also no assessment of measurement error in the reliability analysis or determination of whether or not the study sample (ordinary people rather than patients with CAI were selected) used to assess reliability was appropriate. Another study on CAIT-C’s cutoff scores was reported, but it does not involve the Psychometric Assessments of CAIT-C^48^. Therefore, it is necessary to conduct a more accurate and comprehensive study on the cross-cultural compilation of CAIT in Chinese.

Before discussing the results, the limitations of this study deserve attention. First, China's population may not be fully represented because the sample size is limited. Second, considering translation, the language we use is simplified Chinese, as is the official language. However, as a multiethnic country, many ethnic groups in China have their own languages, such as Hong Kong, Macao, Xinjiang, and the Tibetan Plateau. Hence, in the survey, ethnic cultural differences deserve attention. Last, there was some loss of participants due to exclusion criteria and loss of follow-up, but the overall sample appears to be adequately powered based on the results.

The process of intercultural adaptation and translation is relatively smooth in this study. We believe that the original version of the CAIT project is suitable for the Chinese cultural background. Therefore, we have not adapted the content of questions, which may also benefit from the easy-to-understand advantages of CAIT^21^.

The CAIT-C had good internal consistency (Cronbach’s α = 0.845–0.878), and its Cronbach’s α was slightly higher than that in the original version and other language versions^12,15,21–25^. Simultaneously, we found that Cronbach’s α of CAIT-C would be slightly higher (0.877 and 0.878) when item 8 or item 9 was removed, which also appeared in the Korean version and the Persian version^20,22^. The correlations between the scores of the two items and the total score were the weakest (*r*_s_ = 0.503–0.537). This might be because item 8 and item 9 were set in the hypothesis context (“roll over on ankle”), while other items were about the daily life of the patient, which caused differences in the same patient responding to these items. Good test–retest reliability (ICC = 0.930) is reflected in the CAIT-C, which is consistent with the results of similar studies (Table 3). Additionally, we consider that the assessment of the CAIT-C test–retest reliability as more appropriate using a week as the time interval because the patient is less prone to forget the specific answers in the previous questionnaire within a week, and the patient’s functional status and daily life would not dramatically change in 1 week. MDC and low values for measurement error mean that small clinical changes and individual-level changes can be detected at the population level by CAIT-C.

There was no floor effect or ceiling effect in CAIT-C. The evaluation of three experts also authenticated that the CAIT-C items were well correlated with the patient’s prognosis and CAI patients. In addition, due to the easy-to-understand advantage of CAIT-C, there were no missed responses in any returned questionnaires. Based on the above objective results, and the good feedback from patients who filled out the questionnaire, the CAIT-C had good content validity.

In other cross-cultural adaptation studies on CAIT, except for the Dutch version^21^, the remaining versions all evaluated the criterion validity of CAIT^20,22–25^. However, in light of the COSMIN list (consensus-based Standards for the selection of health status Measurement INstruments), which is a consistency-based checklist for assessing the methodological quality of the measurement attributes of the health measurement instruments based on an international Delphi study, the “criterion validity” was defined as the degree of a PROMs instrument that reflects the degree of "gold standard", in 2010^43^. The standard used should be reasonably considered the "gold standard", but the Delphy group agreed that there was no gold standard for PROMS instruments^43^. The “hypotheses testing” for evaluating the so-called “criterion validity” in other cross-culture adaptation studies was the method for assessing the construct validity of CAIT. By hypothesis testing, the correlations between the CAIT-C and the subscales of the SF-36 and FAAM in this study were the same as that of our previous hypothesis, meaning that the CAIT-C has good construct validity. The CAIT-C had the strongest correlations with the two subscales of the FAAM. Although the FAAM is not a disease-specific scale for patients with CAI, it mainly concerns the functional status of the patient’s foot and ankle (region-specific scale), such as CAIT. Therefore, the objective of FAAM items is very close to that of CAIT. In addition, the CAIT-C had weak correlations with the mental subscales of the SF-36, but the correlation still existed (*P* < 0.05), indicating that the functional status of the foot and ankle in CAI patients would affect their psychological states.

One of the important factors in determining whether the scale can be used in prospective clinical research is the quality of the scale’s responsiveness. In this study, CAIT-C showed good responsiveness, which means that CAIT-C can be sensitive to changes in the functional condition of patients after systemic physicotherapy. Compared with related studies, the ES value of this study was slightly higher (ES = 0.69–1.07)^12,24,25^. This might be because patients in this study received 8 weeks of physicotherapy, and the treatment period in other studies was shorter (3–4 weeks), which led to certain differences in the degree of improvement in the patient’s functional status.

## Conclusions

In summary, we successfully translated CAIT into Chinese. After verification, the version was easy to use and has good responsiveness, reliability, and validity. Hence, we advise that CAIT-C be used in assessing the functional condition of Chinese CAI patients in related clinical work or clinical studies to help researchers or doctors collect the necessary data.
