# Author Correction: Development of a valid Chinese version of the Cumberland Ankle Instability Tool in Chinese-speaking patients with chronic ankle instability disorders

**DOI:** 10.1038/s41598-021-97504-z

**Published:** 2021-09-07

**Authors:** Wei Wang, Dongfa Liao, Xia Kang, Wei Zheng, Wei Xu, Song Chen, Qingyun Xie

**Affiliations:** Department of Orthopedics, The General Hospital of Western Theater Command, Tianhui Road 270, Chengdu City, 610000 People’s Republic of China

Correction to: *Scientific Reports* 10.1038/s41598-021-87848-x, published online 07 May 2021

The original version of this Article contained a repeated error in the Methods section, under the subheading ‘Patients and data acquisition’, and in the Results section, under the subheading ‘Patients’, where

“February 2016 to March 2017”

now reads:

“February 2016 to March 2018”

The original Article has been corrected.

